# Supplementary material for: Leucine and Arginine Availability Modulate Mouse Embryonic Stem Cell Proliferation and Metabolism
Source: Int J Mol Sci. 2022 Nov 18;23(22):14286. doi: 10.3390/ijms232214286 (PMC9694364; doi:10.3390/ijms232214286)

Supplementary material

Leucine and Arginine Availability Modulate Mouse Embryonic Stem Cell Proliferation and Metabolism

Bibiana Correia [1,2], Maria Inês Sousa [1,2], Ana Filipa Branco [2], Ana Sofia Rodrigues [2] and João Ramalho-Santos [1,2,*]

[1] Department of Life Sciences, University of Coimbra, Calçada Martim de Freitas, 3000-456 Coimbra, Portugal
[2] CNC—Center for Neuroscience and Cell Biology, CIBB, University of Coimbra, Azinhaga de Santa Comba, Polo 3, 3000-354 Coimbra, Portugal
* Correspondence: jramalho@uc.pt

Appendix SA. Original blot membranes of c-MYC labeling and its respective CALNEXIN

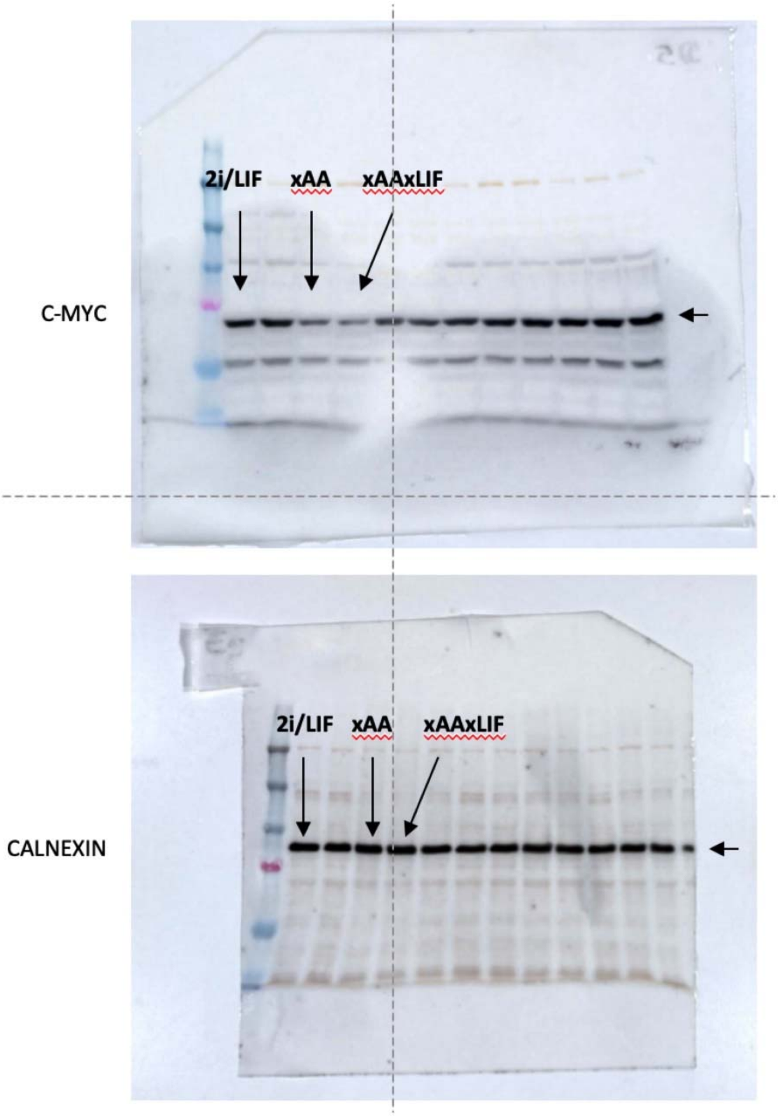

Appendix SB. Original blot membranes of COXIV labeling and its respective CALNEXIN.

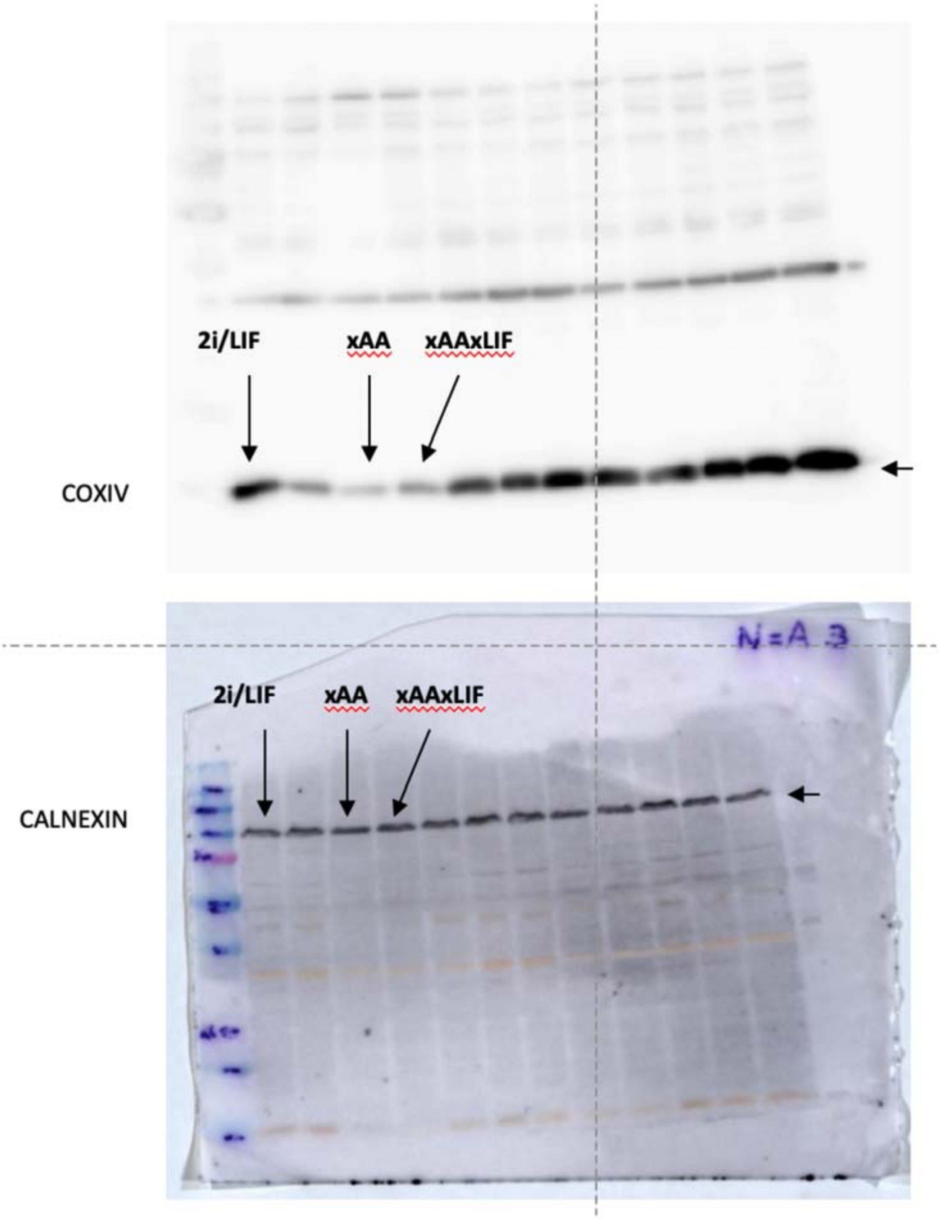

Supplement: Supplementary file 1 [file ijms-23-14286-s001.zip › ijms-2023859-supplementary.pdf]
